# Investigating the effect of mouth guard use on aerobic performance in amateur boxers

**DOI:** 10.1002/cre2.422

**Published:** 2021-03-29

**Authors:** Irfan Ahmed, Courtney Kipps, Peter Fine

**Affiliations:** ^1^ Institute of Sport, Exercise and Health University College London London UK; ^2^ University College London Eastman Dental Institute London UK

**Keywords:** mouthguard, contact sport, dental trauma, soft tissue

## Abstract

**Objectives:**

To assess if wearing a mouth guard impacts maximal aerobic capacity in amateur boxers.

**Materials and Methods:**

A prospective crossover cohort (pilot) study was conducted to assess maximal aerobic capacity in amateur boxers using the 20 m multi stage fitness test (MSFT). Two primary outcomes measures were recorded: (1) the maximum oxygen uptake (peak VO_2_—mL/kg/min) and (2) distance run (meters—m). Thirteen amateur boxers completed the MSFT 7 days apart under control (no mouth guard—C) and intervention conditions (mouth guard—MG). Participants also submitted data on height, weight, type of mouth guard and Rate of Perceived Exertion (RPE) during the test.

**Statistics:**

Paired *T*‐test.

**Results:**

Mouth guard use was shown to reduce peak VO_2_ and distance run during the 20 m MSFT from 56.31 to 54.12 mL/kg/min and 2572 to 2380 m respectively (*p* < 0.05). Twelve out of 13 participants wore a Boil & Bite mouthguard and recorded lower peak VO_2_ scores (−4.38%) when wearing a mouth guard compared to control conditions, (Mean = −2.46 mL/kg/min, Range of decrease = 4.2–0.9 mL/kg/min; *p* < 0.05). Ten participants submitted data on RPE—One participant with a custom made mouthguard reported no change in RPE in mouthguard conditions, while nine participants reported an average (+30.5%) increase in mean RPE in Boil & Bite mouthguard conditions compared to control conditions.

**Conclusions:**

Boil & Bite mouth guard use was shown to significantly reduce aerobic performance in amateur boxers and increase the perceived rate of exertion during the 20 m MSFT.

## 1 INTRODUCTION

The first “sports” mouth guard was designed by a London Dentist called Woolf Krause in 1890. He primarily designed the first mouth guard to act as a rudimentary “gum shield” and protect professional boxers from soft tissue (lip) lacerations. Since then advances in sports dentistry have allowed for customized mouth guards to be designed, with better shock absorption and injury prevention properties. A recent meta‐analysis has shown that non‐mouth guard use in sports is associated with a 1.6–1.9 increased risk of orofacial trauma (OFT; Knapik et al., 2007). It remains to be seen however, if wearing mouth guards may have a secondary impact on athletic performance; with some small studies showing a negative impact (Delaney & Montgomery, 2005) and others none (Garner et al., 2011).

Whilst the debate continues regarding performance and mouth guard use, it has been left to individual sports to determine whether they wish to mandate their use. Governing bodies across all sports, have adopted varying approaches: with some mandating use at a national level by comprehensive medical guidance, and other adopting a local or regional approach to enforcement. To enforce the mandatory use of mouthguard the amateur international boxing association (AIBA) has issued comprehensive medical guidance stating that a mouth guard should be worn to “fit exactly and comfortably” during competition (AIBA Rules and Guidelines—AIBA, 2019).

During an adult amateur boxing contest, participants will be subject to three rounds of intense physical activity of between 2 and 3 min, followed by a 1‐min rest period. Amateur boxers will need to rely on skill, physical conditioning, and contributions from aerobic and anaerobic respiration (Davis et al., 2014) to evade their opponent and land scoring punches. In order to win, well trained boxers will be able to pace themselves through a contest to ensure they have sufficient cardiovascular, respiratory and energy reserves throughout.

To advance the discussion on whether mouth guard use should be mandated in professional and amateur “at risk” sports. We tested the null hypothesis that mouth guard use has no effect on the maximal aerobic capacity of well‐trained amateur boxers who routinely wear them.

### 1.1 Orofacial injuries in sport

Whilst the exact incidence of OFT injuries in sport is not accurately known, expert opinion has advocated for their use in primary prevention of injury. The annual cost of treatment for all dental trauma is significant and thought to be ($2–5 million/1 million inhabitants) in the United States (Andersson, 2013). Data collected from over 100 pediatric Emergency Departments has shown that children are particularly susceptible, with sports and recreational activities accounting for 45.6% of all non‐fatal OFT injuries seen (Kamboj et al., 2019).

A questionnaire survey of 1189 athletes across six high‐risk sports reported that 28.8% of respondents had sustained at least one dental related injury during their career (Ferrari & de Medeiros, 2002). Further single sports studies have shown that the incidence of at least one OFT during a sports career to be: 23% (Taekwondo), 11% (Soccer), 23.5% (Muay Thai), 100% (triathlon) and 73.6% (Boxing; Aljohani et al., 2017; Andrade et al., 2010; Chatrchaiwiwatana, 2016; Qudeimat et al., 2019). These studies based on self‐reported injuries show that OFT injuries are a common occurrence in sports; the immediate consequences of which may require a player to be withdrawn from competition, lose time from training or be left with long term cosmetic consequences.

The American Dental association has therefore issued guidance recommending that well‐fitting mouth guards are worn in 29 sports or recreational activities where participants are at risk of: “injury to the teeth, jaw and oral soft tissues (mouth, lip, tongue, or inner lining of the cheeks”. Despite the recommendations from dental professionals, only a limited number of sports have mandated their use centrally; instead leaving the decision up to local sports administrators, schools, coaches and individuals to enforce. Further barrier to their universal widespread use include discomfort, restriction to talking and breathing concerns from athletes (Matalon et al., 2008).

### 1.2 Mouth guard design

Mouth guards are designed to fit over the occlusal surfaces of the teeth and gingivae of the maxilla or mandible. Upper maxillary mouth guards are recommended in amateur boxing, due to this being the most common location of injury during competition. The cost of mouth guards can vary significantly from £5–10 for Stock or Boil & Bite mouth guards bought over the counter to £50–200 from custom made mouth guard from a dental professional.

Stock mouth guards—These mouth guards are made of polyurethane, co‐polymer, vinyl acetate or ethylene and are bought ready made over the counter. As they are not molded to the patient, they require inter‐maxillary pressure to maintain their position, and can be liable to poor fitting.

Boil and Bite (B&B) mouth guard—Mouth formed or Boil & Bite guards are made of a thermoplastic and molded to the athlete's teeth and gingivae after immersion in hot water. They require the athlete to apply suitable pressure to mold appropriately, but as a consequence their thickness can vary depending on the impression made.

Custom mouth guards—These are the most expensive mouth guards and are made from an impression of the athlete's teeth. The materials and composition of the mouth guard can vary, with several companies offering laminate composites to suit the individual and demands of each sport.

### 1.3 The role of mouth guards in performance

Several small studies have looked at the impact of wearing mouth guards, with regards to how the altered temporomandibular joint position and obstruction to the anatomical airway, effects ventilation. All three type of mouth guards are associated with a reduction in forced expiratory volume in 1 s (FEV_1_) and functional vital capacity (FVC; Caneppele et al., 2017) at rest, but few studies have looked at the impact this has on prolonged maximal aerobic exercise. Athletes who are performing at sub maximal effort can compensate for this reduction by increasing their respiratory rate and tidal volume, if they have sufficient reserves.

Previous studies using approximations from exhaled respiratory gases have shown that amateur boxers will rely on the majority of their energy from aerobic respiration (77%; Davis et al., 2011). In addition to this VO_2_ max scores in elite national (Smith, 2006) and amateur levels (Bruzas et al., 2014) boxers have consistently shown high aerobic capacities (Smith, 2006).

### 1.4 Aerobic versus anaerobic exercise

Total energy production during exercise is a marker of fitness relying on elements of both “anaerobic” and “aerobic” respiration. The anaerobic pathway is responsible for producing energy without requiring oxygen (Chamari & Padulo, 2015). As this pathway does not utilize oxygen, the use of mouth guards is not thought to adversely effect it directly. Aerobic respiration however is dependent on oxygen and requires athletes to have sufficient cardiovascular and respiratory reserves to maintain energy production.

The relative contributions of these two pathways changes with a trend for an increasing reliance on aerobic respiration as the duration of exercise increases. Studies looking at runners subjected to a “maximal” steady treadmill protocol have shown that after 30 s, aerobic respiration is the predominant pathway for energy production (Spencer & Gastin, 2001; Table 1).

**TABLE 1:** Classification for all out maximal aerobic exercise efforts

Duration (s)	Description	Predominant source of energy production	Requires oxygen
1–6	Explosive efforts	Creatinine kinase + anaerobic	−
6–60	High intensity efforts	Anaerobic + aerobic	+
>60	Endurance intensive efforts	Aerobic predominantly	+++

## 2 METHODS

Recruitment—Members of the Cambridge University Amateur Boxing club were invited to take part in this pilot study via an email advert. Informed consent was gained prior and partition in the study was voluntary.

Ethics—Ethical approval for the study was granted by University College London (approval number 14715/001).

Inclusion Criteria—In order to be eligible for the study all participants had to have trained for a minimum of 8 weeks with an upper (maxillary) mouth guard and completed the 20 m MSFT at least once before.

Exclusion Criteria—Athletes who were injured or suffering from illness were excluded.

Data collected—Maximal aerobic capacity was assessed using the 20 m multi stage fitness test (MSFT). Two primary outcomes measure were recorded: (1) the maximum oxygen uptake (peak VO_2_—mL/kg/min) and (2) distance run (meters—m). After each test, the participants were also asked to submit Borg scale scores for Rate of Perceived Exertion (RPE, scale 0–10). Height and weight data were collected from each participant.

Testing protocol—The order of the tests was determined by a coin toss on day 1, neither the participants nor test administrator knew which test condition was to be performed prior. Test 1 was performed in control conditions and test 2 in mouthguard conditions.

Statistics—Results were analyzed on Microsoft Excel (San Diego, CA) using a paired *T*‐test.

Sample size calculation—A minimum sample size of eight pairs (Dhand & Khatkar, 2014) was calculated on there being an expected standard deviation of the paired differences of 3.1 (mL/kg/min; Aandstad et al., 2011) and an expected mean of the paired differences of 4 (mL/kg/min).

### 2.1 20 m‐Multistage fitness test

All participants completed the 20 m MSFT at the Cambridge University Sports Centre. The test is associated with a high repeat test reliability and provides an indirect measure of peak VO_2_ (mL/kg/min; Cooper, 2005). The following variables were standardized and maintained in both test conditions: floor surface, footwear, temperature, time of test and a 24 h rest period prior and the test operator. All participants completed the test prior to the weekly circuit training session to ensure they had adequate rest.

## 3 RESULTS

Eleven males and two female participants took part in the study with a mean age of 22.77 years. The mean average height and weight of participants was 176.69 cm and 71.85 kg, respectively. Twelve participants used a Boil & Bite mouth guard and one participant used a custom‐made mouth guard in intervention (mouthguard) testing conditions.

**FIGURE 1:**
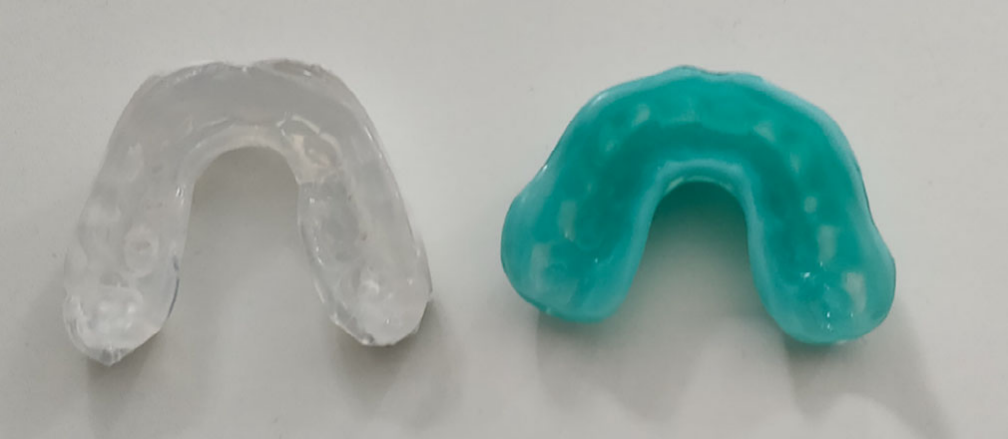
Boil & Bite mouthguard versus custom made laminate design mouthguard. Mouthguard type Boil & Bite (left ‐ clear colour) & custom made (right‐ light green colour)

The average peak VO_2_ score in the control conditions was significantly higher (56.34 mL/kg/min) than the mouth guard condition (54.12 mL/kg/min; *p* < 0.05; Figure 1). There was an average reduction in peak VO_2_ score of 4.31% (mean = −2.43 mL/kg/min, Range 4.2–0.9 mL/kg/min) across all participants. A subgroup analysis of Boil & Bite mouthguard users (*n* = 12) showed that Boil & Bite mouthguard use was associated with a (4.37%) reduction in peak VO_2_ when wearing a mouth guard compared to control conditions (*p* < 0.05; Table 2).

**TABLE 2:** Mouth guard versus control conditions—Paired *t*‐test analysis of distance run (m), peak VO_2_ and Borg rate of perceived exertion

Results for all mouthguard users (n = 13)				
	Control (C)	Mouth guard (MG)	Average % change	*p*‐Value
Distance run (m) mean ± SD	2572 ± 408	2380 ± 366	−7.46	8.54 × 10^−6^
VO_2_ max (mL/min/kg) mean ± SD	56.34 ± 5.68	54.12 ± 5.19	−4.31	2.87 × 10^−6^

Subgroup analysis of results for Boil & Bite mouthguard users only (*n* = 12)				
	Control (C)	Boil & Bite mouthguard	Average % change	*p*‐Value
VO_2_ max (mL/min/kg) mean ± SD	56.31 ± 5.91	53.85 ± 5.39	−4.37	9.13 × 10^–6^
Rate of perceived exertion (Borg scale) Mean ± SD	6.00 + 1.73	7.83 ± 1.87	+30.50	0.0073

Ten participants submitted data on RPE—One participant with a custom made mouthguard reported no change in RPE in mouthguard conditions, while nine participants reported a +30.5% increase in mean RPE in Boil & Bite mouthguard conditions compared to control conditions.

**FIGURE 2:**
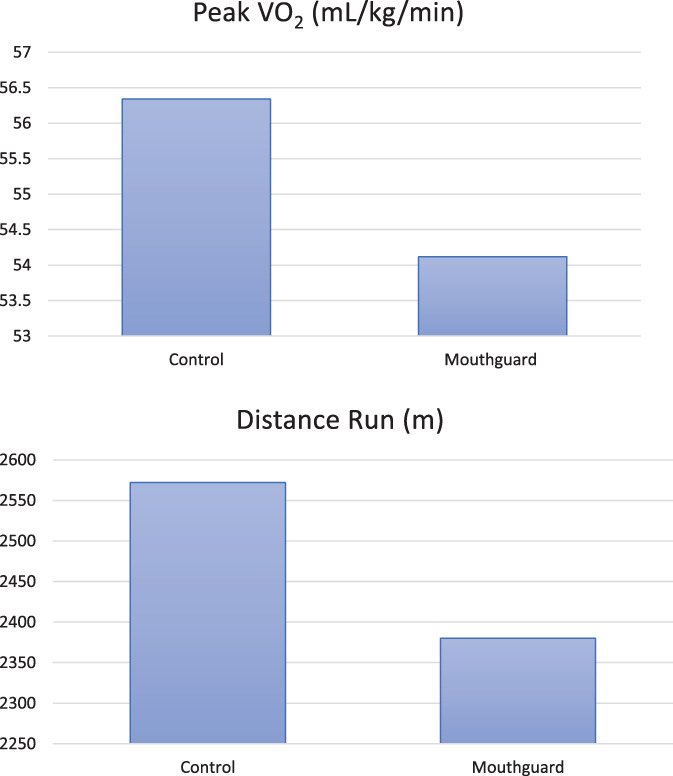
Mean distance run (m) and peak V0_2_ achieved in the control group versus mouth guard group

All 13 participants completed the standard 20‐m MSFT in both conditions to satisfy the crossover trial. Mean distance achieved in the MG group (2380 m) was significantly lower than the control conditions (2572 m; *p* < 0.05; Figure 2).

## 4 DISCUSSION

The main aim of this pilot study was to assess if mouth guards effect aerobic performance in amateur boxers performing the 20‐m MSFT. The results of this study suggest that mouth guard use does reduce average (mean) aerobic performance (−2.22 mL/kg/min) but the results of this study must be interpreted in the context of the type of mouth guards worn and study design. Twelve of the 13 participants in the study wore Boil & Bite mouth guards, with only one wearing a custom‐made mouth guard. The results of this cross over study therefore predominantly reflect an analysis of how Boil & Bite mouth guards effect maximal aerobic performance.

Whilst Boil & Bite mouth guards are molded to the individual athlete, the quality of the fit is determined by the technique of the individual. Previous studies of boil and bite mouth guards have shown that they inhibit athlete's ability to talk, are subject to discomfort and poor retention (Matalon et al., 2008). Nine participants who submitted data reported a higher (+30.5%) rating of perceived exertion in Boil & Bite mouth guard and one participant who wore a custom‐made mouth guard reported no difference in RPE. An analysis of whether custom made mouth guards improve comfort was beyond the scope of this study, but previous studies have shown that custom made mouth guards are associated with a better fit and reduced discomfort when worn (Duarte‐Pereira et al., 2008).

Our Pilot study demonstrated that mouth guards are associated with a small reduction in maximal aerobic performance (−4.31%), when compared to control conditions. Previous studies on mouth guards have shown conflicting results with some suggesting that mouth guards do reduce aerobic performance (Delaney & Montgomery, 2005; El‐Ashker & El‐Ashker, 2015), and others suggesting that they do not when custom mouth guards are used (Garner et al., 2011; Gebauer et al., 2011; Kececi et al., 2005).

Caneppele et al. (2017) performed a systematic review of 14 studies that assessed markers of aerobic performance (VO_2_ max) with mouth guards; but found great variability in the type of aerobic testing protocol used. Many of the studies used participants from sports where athletes would not be expected to achieve maximal aerobic capacity for prolonged periods or did not utilize true maximal exercise testing. In addition seven studies did not detail if they randomized the sequence of tests or what steps they took to minimize any potential order effect (Caneppele et al., 2017).

It is well established that mouth guards act as a physical obstruction to the airway and reduce markers of ventilation at rest. Less is known about how athletes may be able to adapt to this or alter their breathing pattern during maximal aerobic exercise (von Arx et al., 2008). We did not assess ventilation during exercise but hypothesize that this restriction to ventilation only becomes significant at maximal aerobic efforts when athletes approach their maximum minute ventilation (L/min). Previous studies with submaximal testing protocols, may not have achieved this threshold and therefore may have underestimated the effect of mouthguards on performance.

Francis et al. took direct gas measurements of athletes during exercise and proposed that wearing mouth guards may mimic purse lip breathing (PLB) patterns (Francis & Brasher, 1991). These breathing patterns have been shown to, improve the efficiency and reduce the metabolic work of breathing in clinical populations of chronic obstructive pulmonary disease (COPD) patients. Further studies directly measuring volumes of expired gases, will be needed to confirm if this compensatory benefit applies to healthy athletes performing maximal aerobic exercise.

The 20 m multistage fitness test MSFT is a well validated test of maximal aerobic performance but only provides an indirect measurement of peak VO_2_. The results of this pilot study suggest that Boil & Bite mouth guards do reduce estimated peak VO_2_, however this must be balanced against the known risk of OMF trauma. A meta‐analysis of 14 studies has shown that non‐mouth guard use is associated with a 1.6–1.9 increased risk of OMF trauma across all sports (Knapik et al., 2007). Further robust epidemiological studies will be required to work out the exact incidence of OMF trauma in boxing but given the nature of the sport it is expected to be at least as common as other combat sports (Aljohani et al., 2017; Andrade et al., 2010; Chatrchaiwiwatana, 2016; Qudeimat et al., 2019).

### 4.1 Limitations

The main limitations of this pilot study were that we did not standardize the mouthguard type and were only able to indirectly measure estimated Peak VO_2,_ via the MSFT. We plan to perform further studies with custom made (dental) mouthguards, and directly measure maximal oxygen uptake (VO_2_ max—mL/kg/min), lactate thresholds and markers of ventilation during a cardio pulmonary exercise test (CPET). This will allow us to determine in more detail if mouthguards impact maximal or submaximal efforts and provide us with a mechanism of how this may occur, from athletes cardiac, respiratory and metabolic response.

### 4.2 Implementing mouthguard use for safety

Individual sports must decide if the reduction in OFT, outweighs any modest impact on performance or discomfort for athletes. It has been proposed that in under 16's sport the continued safe participation of athletes, outweighs any small reduction in performance observed and other sports should consider mandating for their use at a national or organization level (Ahmed & Fine, 2021). Amateur and professional boxing has shown that comprehensive medical guidance can help to promote the mandatory use of mouthguards, and that this is not a barrier to athletes wanting to participants at all levels of competition.

We therefore suggested that as per the American Dental Association of America guidance (Mouth Guards, 2019): athletes at risk of injury should wear a well fitted mouth guard. Whilst the official guidance stops short of recommending one mouth guard type over another, the protective properties, relative cost and benefit must be taken into consideration when counseling athletes.

In order to reduce discomfort during training and competition, athletes may consider wearing mouthguards for a period of acclimatization, and using break periods in the sport such as between rounds, tactical stoppages or during half time to remove the mouthguard when not needed.

## 5 CONCLUSION

The results of this study demonstrate that Boil & Bite mouth guard use is associated with a small but statistically significant reduction in peak VO_2_ (mean = −2.46 mL/kg/min) when measured in amateur boxers completing the 20 m MSFT (*p* < 0.05). In addition to this, participants reported that wearing a Boil & Bite mouth guard increased the rate of perceived exertion by 30.5% during the 20 m MSFT (RPE—Borg scale; *p* < 0.05).

## CONFLICT OF INTEREST

No conflicts of interest to declare.

## AUTHOR CONTRIBUTIONS

I.A. and P.F. conceived and designed the study. I.A. drafted the manuscript, with supervision from C.K. and P.F. during the course of an MSc. I.A conducted the statistical analysis and interpretation of the data. All authors have read and approved the final manuscript.

## FUNDING DECLARATION

No relevant declarations. The manuscript was prepared as part of the academic element of an MSc course.

## Data Availability

In accordance with the journals policy on Data Sharing and Data Accessibility The data that support the findings of this study are available from the corresponding author upon reasonable request. Dr Irfan Ahmed On behalf of the manuscript authors.
